# Administration of a Postbiotic Causes Immunomodulatory Responses in Broiler Gut and Reduces Disease Pathogenesis Following Challenge

**DOI:** 10.3390/microorganisms7080268

**Published:** 2019-08-17

**Authors:** Casey N. Johnson, Michael H. Kogut, Kenneth Genovese, Haiqi He, Steve Kazemi, Ryan J. Arsenault

**Affiliations:** 1 Department of Animal and Food Sciences, University of Delaware, Newark, DE 19716, USA; 2 Southern Plains Agricultural Research Center, USDA ARS, College Station, TX 77845, USA; 3 Pure Cultures Inc., Denver, CO 80216, USA

**Keywords:** kinome, postbiotic, gut, *Clostridium perfringens*, chicken, immunomodulatory

## Abstract

With the reemergence of poultry diseases such as necrotic enteritis following the restriction of in-feed antibiotics, the search for antibiotic alternatives has become critically important. Postbiotics are non-viable bacterial products or metabolic byproducts from probiotic microorganisms that have positive effects on the host or microbiota. These are a promising alternative to antibiotics. Here, we describe the mechanism of action of a postbiotic in the context of a *Clostridium perfringens* (*C. perfringens*) challenge model. By using performance measurements and a peptide array kinome analysis, we describe the kinotypes and signal transduction changes elicited by the postbiotic with and without *C. perfringens* challenge. The postbiotic improves lesion scores, *C. perfringens* counts and mortality compared to challenge groups without the postbiotic, and it improves weight gain in the most severely challenged birds. The postbiotic predominantly affects the innate immune response and appears immunomodulatory. In the context of infection, it reduces the proinflammatory responses and generates a homeostatic-like response. This postbiotic is a viable alternative to antibiotics to improve poultry health in the context of *C. perfringens* pathogen challenge.

## 1. Introduction

Commercial poultry is one of the most efficient sources of animal-protein available today [1]. With a growing worldwide human population and a shift within the population to a diet higher in animal protein, the production of safe and efficiently produced food-animals has never been more important [2]. At the same time, there has been an increase in the restrictions placed on available disease treatments for chicken and the management of chicken production [3]. The federal government has begun enforcing the Food and Drug Administration Veterinary Feed Directive in 2017 that is significantly reducing the availability and usage of antibiotics as growth promoters in farm animals [4]. Antibiotics are useful tools that prevent/treat disease, improve feed conversion, improve animal health/welfare and reduce carbon footprint [5]. Viable alternatives to antibiotics are needed. As a result, finding alternatives to previous growth promoting and health enhancing practices is a major research enterprise. The challenge for poultry producers is to feed the world while following the regulatory mandates of the local jurisdictions where the birds are raised. One promising avenue for the enhancement of bird growth and health are “biotic” feed additives, probiotics, prebiotics and postbiotics [6]. Probiotics, more commonly referred to in animal production as direct fed microbials, are live bacterial cultures that take up residence in the animal gut and, ideally, provide a beneficial function. Prebiotics are feed components not digestible by the host and are specifically selected to foster the growth of beneficial gut bacteria. Finally, postbiotics are fed products that are generally produced by beneficial gut microbes and have a positive impact on host health. 

In the intestine, the number of genomes and biochemical reactions within the microbiota are greater than those of the host and significantly impact many aspects of host health, metabolism, immunity, development and behavior, while microbial imbalance, referred to as dysbiosis, is associated with disease [7]. A critical and well understood function of the gut microbiota is the metabolism of host-indigestible feed ingredients. This ability of the microbiome significantly enhances the energy utilization potential of feed by the host. The microbiota and the metabolites they produce also have a profound effect on the signal transduction of the host and are often regulators of host response [8,9]. Postbiotics are substances produced by these metabolic processes in bacteria. They are effectively probiotic metabolic byproducts and non-viable bacterial products. The various metabolic byproducts produced as postbiotics include: Short chain fatty acids, bacteriocins, functional peptides and proteins [6,10]. The immune system–microbiota cross talk is necessary for the proper development and functioning of immunity. Recent studies have uncovered a major role for microbial metabolites in the regulation of the immune system [9]. 

A major challenge when studying feed additives is determining the mode of action and efficacy. This is especially true for those products that may enhance growth under certain production conditions or that impact the immune system upon challenge or stress. Often, statistically significant data are difficult to generate in studies performed in well-controlled conditions such as a university research farm. These studies do not mimic the wide variety of conditions and challenges of a normal production situation. Our approach has been to determine the molecular mechanism of action of feed interventions by studying the relevant host tissue itself from a proteomic perspective [11]. This molecular proteomic approach has two major advantages. One advantage is that in considering the change in protein function, we do not have to make assumptions generally made in genomic or transcriptomic data analysis, namely that the gene expression changes result in true phenotypic alterations. The second advantage is that an effective feed intervention will elicit a proteomic change based on its interaction with either the gut microbiome or the host tissue. Even if this change is not detectable in gross measurements, such as feed-conversion ratio or daily weight gain, it is detectible at the level of changes to the function of proteins, cells, and tissue. 

The technique our group uses to measure these proteomic changes is the species-specific kinome peptide array [12,13]. This technology is designed for the species of interest—in this case chicken—and measures the activity of kinase enzymes within a tissue sample. Kinases are enzymes which phosphorylate protein, altering their function. Phosphorylation is the major post-translational modification carried out by host cells, and it has an impact on nearly every physiological function [14]. The array is specifically designed to measure changes in innate and adaptive immunity, carbohydrate, protein and fatty acid metabolism, and stress responses [15].

The suspension of antimicrobial growth promoters and ionophore coccidiostats has resulted in a reemergence of necrotic enteritis (NE), a severe *Clostridium perfringens* (*C. perfringens*)-induced disease that results in devastating clinical disease in chickens [16]. Historically, antibiotics have been used to control *C. perfringens*-induced necrotic enteritis in poultry. With growing concern about antibiotic resistance, poultry producers are looking for alternatives to combat this disease that is estimated to cost the world poultry industry up to $6 billion annually [17]. 

The purpose of this project was to evaluate postbiotic water additives from a proprietary pure probiotic culture of three lactic acid bacteria that may improve the health and welfare of the bird. We used coccidiosis vaccine and *C. perfringens* infection model as the challenge to bird health. Results from this study show that the postbiotic product resulted in detectable weight gain and reduced lesion scores compared to challenged groups. Changes in the kinome profile of the gut tissue of broiler birds, with and without pathogenic challenge, were detected. These changes were predominantly in the jejunum and centered on immune-related activities. Based on the comparison with the challenge groups, the nature of the immunological change was to modulate the immune response and dampen pathological signaling induced by the *C. perfringens* challenge, keeping the broiler gut in a state of homeostasis. The postbiotic appears to predominantly impact the innate immune system—the first line of defense and the branch of the immune system most quickly induced by a pathogen. The mechanism of this impact on innate immunity is centered on the phosphoinositide 3-kinase—protein kinase B (PI3K-Akt) signaling pathway and the decreased phosphorylation of key signaling intermediates. By understanding the mechanism of action, strategic feed interventions can be designed to better protect poultry health and reduce disease pathogenesis.

## 2. Materials and Methods 

### 2.1. Bird Trial

The study was conducted at the Southern Plains Agricultural Research Center, Agricultural Research Service, United States Department of Agriculture and was approved by Animal Care and Use Committee at the Southern Plains Agricultural Research Center. All experiments were conducted according to guidelines established by the United States Department of Agriculture (USDA) Animal Care and Use Committee, which operates in accordance with established principles (National Research Council Guide for the Care and Use of Laboratory Animals 2011). The protocol was approved by the acting USDA Plains Area Animal Care and Use Committee that operates at the location where the experiments were carried out, Institutional Animal Care and Use Committee (IACUC) No. 2018-006.

A basal industry type broiler starter diet was prepared to meet or exceed the 1994 National Research Council’s Nutrient Requirements of Poultry. These diets were fed as crumbled pellets. Feed was kept in closed plastic/rubber containers and visually inspected every 6–9 days for pest contamination. The water was observed daily to assure the proper function of the automatic delivery system. 

The study consisted of two replicates for a total of 400 broiler chicks. For a single replicate, a total of 200 broiler chicks were distributed among 8 floor pens lined with pine wood shavings (litter). A total of 8 treatments were randomly assigned to pens, and the birds were allowed free access to a commercial formulated broiler feed. Water (untreated or containing postbiotic product as appropriate) was available at all times throughout the trial. For the second replicate, two hundred and fifty fertile eggs (18-days-old) were transported from a commercial hatchery to the USDA facility for hatching. Once eggs hatched, 200 birds were moved to the rearing house on USDA facility. Group body weights were taken on days 1, 14, and 21. On day 14, all birds were orally gavaged with a 10 X dose of the coccidia vaccine (Coccivac B52, Kenilworth, NJ, USA). On days 17–19, three birds from the negative control, postbiotic negative control, Coccivac control, and Coccivac plus postbiotic were necropsied and had gut tissue samples taken, while the remaining birds were orally gavaged with 1–3 mLs of a liquid thioglycollate medium (Becton Dickinson Co., Sparks, MD, USA) broth culture of *C. perfringens* at 10^7^–10^9^ colony-forming units (cfu)/mL daily for three successive days (days 17–19). On day 21, birds were euthanized, weighed and examined for the degree of presence of necrotic enteritis lesions.

### 2.2. Postbiotic Product From a Proprietary Pure Probiotic Culture

Starting on day 1, postbiotic product was added to water at manufacturer’s recommendations of 1 ounce/gallon of fresh water using plastic watering jugs or 5 gallon buckets hooked up to nipple drinkers until the end of the study except for T1, T3, T4, and T5, which were given water with no metabolite products added (Table 1). Every 4–6 days, water systems were cleaned, and fresh product water mixture was made and provided to the T2, T6, T7, and T8 treatment groups. Pure Cultures, Inc. produced a fermented product containing organic acids produced from a consortium or cocktail containing the following strains: Pediococcus acidilactici* (Agricultural Research Service Culture Collection (NRRL)# B-67717), Lactobacillus reuteri* (NRRL# B-67718), Enterococcus faecium* NRRL# B-67720), and Lactobacillus acidophilus** (NRRL# B-67701) (*proprietary strain to Pure Cultures, Inc.; **non-proprietary strain). Product is trademarked Flock Vitality^TM^. 

### 2.3. Necrotic Enteritis Intestinal Lesion Scoring and Tissue Sampling: 

On day 21, birds were humanly euthanized; all euthanasia procedures followed the guidelines set down in the American Veterinary Medical Association (AVMA) Guidelines for the Euthanasia of Animals. Birds were examined for the degree of presence of necrotic enteritis lesions. All birds were lesion scored by the same person throughout the study, and 12 birds had tissue samples (liver, duodenum, jejunum and ileum) taken for further immunological/microbial testing. To quantitatively measure *C. perfringens*, a section of the small intestine about 15–20 cm in length, cranial to Meckel’s diverticulum, was removed. The sample was placed in 10 mL of anaerobic thioglycollate and stomached for 30 s. A 0.5 mL sample was removed from the stomached material, placed into an anaerobic vial containing 4.5 mL of anaerobic thioglycollate, and placed into an anaerobic chamber. The stomached material was incubated for 24 h at 37 °C and streaked on blood agar. The following day, the plates were examined for the presence of *C. perfringens* colonies. The scoring was based on a 0–4 lesion scoring, with 0 being normal and 4 being the most severe. In trial 2, on days 17–19, three birds from T1, T2, T3, and T7 were euthanized following the AVMA Guidelines for the Euthanasia of Animals. Tissue samples (liver, duodenum, jejunum and ileum) taken on days 17–19 were collected for further immunological/microbial testing. Tissue samples from the duodenum and the jejunum of five birds per experimental group were collected at day 21, flash frozen in liquid nitrogen, and sent to the University of Delaware to conduct kinome analysis using chicken-specific peptide arrays.

### 2.4. Chicken-Specific Immunometabolic Kinome Peptide Array

Biological replicate tissues from 5 birds per group were analyzed by a kinome peptide array. Peptide array protocol was carried out as previously described and summarized below [18]. Briefly, 40 mg of tissue samples were used for the kinome peptide array protocol. Samples were homogenized by a Bead Ruptor homogenizer in 100 uL of a lysis buffer containing protease inhibitors. Homogenized samples were then mixed with an activation mix containing ATP and applied to peptide arrays. Arrays were incubated in a humidity chamber at 37 °C with 5% CO_2_, thus allowing kinases to phosphorylate their target sites. Samples were then washed off the arrays, and a florescent phosphostain was applied. Stains not bound to phosphorylated sites were removed by a destaining process. Arrays were then imaged using a Tecan PowerScanner microarray scanner (Tecan Systems, San Jose, CA, USA) at 532–560 nm with a 580 nm filter to detect dye fluorescence.

Array images were then gridded using GenePix Pro software (Molecular Devices, San Jose, CA, USA), and the spot intensity signal was collected, thus ensuring peptide spots were correctly associated with their phosphorylation sites. Greater intensity fluorescence correlates to greater phosphorylation at the target site.

Fluorescent intensities for treatments were then compared with controls using a data normalization program—Platform for Intelligent, Integrated Kinome Analysis, version 2 (PIIKA2) [19]. The resulting data output was then used in downstream applications such as Search Tool for the Retrieval of Interacting Genes/Proteins (STRING) [20] and Kyoto Encyclopedia of Genes and Genomes (KEGG) [21] databases used to pinpoint changes in protein–protein interactions and signal transduction pathways.

## 3. Results

### 3.1. The Postbiotic Improved Performance Compared to Challenge Groups

The *C. perfringens* colony forming units (cfus), bird weight gain, and lesion scores were measured and combined for each group for each of the two replicate trials (Table 2 and Table 3). In both trials, there was a reduction in cfus of *C. perfringens* in the jejunum (Meckel’s adjacent) when inoculated with *C. perfringens* and treated with the postbiotic compared to just inoculated (9.5 vs. 14.0 × 10^5^ trial 1; 2.3 vs. 5.9 × 10^5^ trial 2). There was also a reduction in cfus of *C. perfringens* when inoculated with *C. perfringens* plus Coccivac plus postbiotic vs. *C. perfringens* plus Coccivac (1.7 vs. 6.2 × 10^5^ Trial 1; 0.5 vs. 7.4 × 10^5^ Trial 2). For weight gain over the 21 days, the postbiotic actually numerically reduced gain compared to *C. perfringens*-inoculated groups except in the *C. perfringens* plus Coccivac and postbiotic vs. *C. perfringens* plus Coccivac (781 vs. 697 g Trial 1; 795 vs. 664 g Trial 2) and the *C. perfringens* plus postbiotic vs. *C. perfringens* alone in Trial 2 (725 vs. 647 g). The postbiotic reduced lesion scores in all inoculated groups, most dramatically in Trial 2 between the *C. perfringens* plus Coccivac and postbiotic group compared to the *C. perfringens* plus postbiotic group (0.41–1.89 score). Between these two groups, mortality was also reduced in Trial 2 from 5/25 challenged birds to 0/25 challenged birds treated with the postbiotic. 

### 3.2. Heatmap and Clustering Results Show the Largest Kinome Impact of Postbiotic Is in the Jejunum

**Figure 1:**
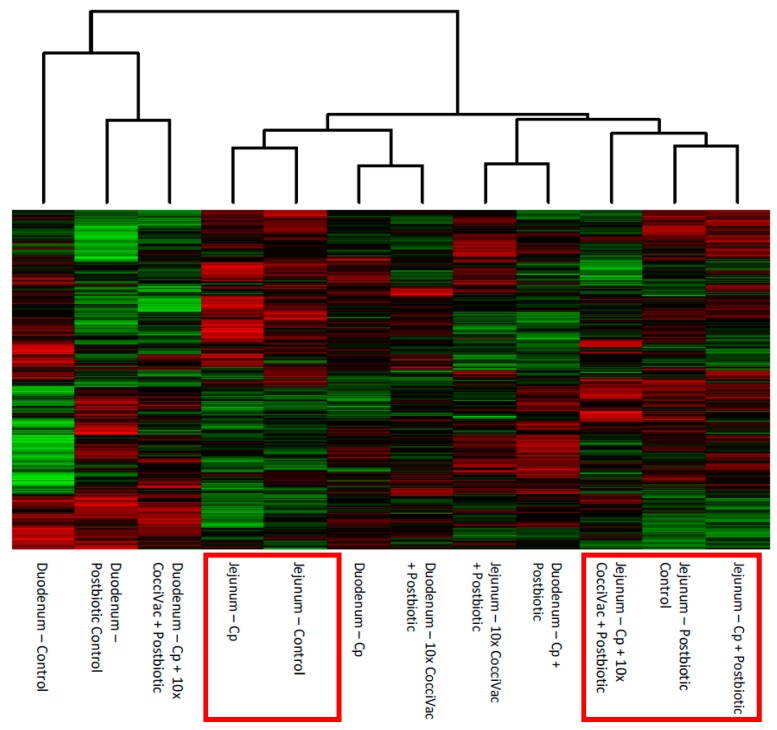
Heatmap and Clustering of Kinome Profiles. The raw kinome signal from the peptide array was input into the custom software package PIIKA 2. PIIKA 2 combines the biological replicates for each treatment and tissue, normalizes the data, and generates a representative kinome profile. Here, the profiles are compared for relative similarity, and a heatmap shows the relative phosphorylation of each peptide on the array. Cp = *C. perfringens*.

Phoshorylation signal results generated from biological replicate samples from five birds per group were combined to generate representative kinome profiles—kinotypes. Kinome profiles were generated for tissue/experimental group combinations. Following data combination and normalization, cluster analysis was performed on the resulting 12 kinotypes (one for each tissue (2) X treatment group (6)). Figure 1 shows the heatmap, with red indicating relative increased phosphorylation, green indicating the decreased phosphorylation of each peptide on the array, and the clustering at the top of the figure displaying the relative similarity between the 12 profiles (X-axis).

An effect of the postbiotic is evident from how the experimental groups clustered. First, the jejunum samples tended to cluster together (Figure 1, red boxes). Second, the jejunum samples from birds not treated with the postbiotic formed a separate cluster (Control and *C. perfringens*-inoculated, left box) from those that were treated (right box). This suggests that the postbiotic treatment had a distinct effect on jejunal tissue, different from the effect of the *C. perfringens* challenge. Overall, the duodenum samples did not seem to develop a particular clustering pattern. The duodenum from postbiotic-treated groups tended to cluster closer to the control samples overall.

**Figure 2:**
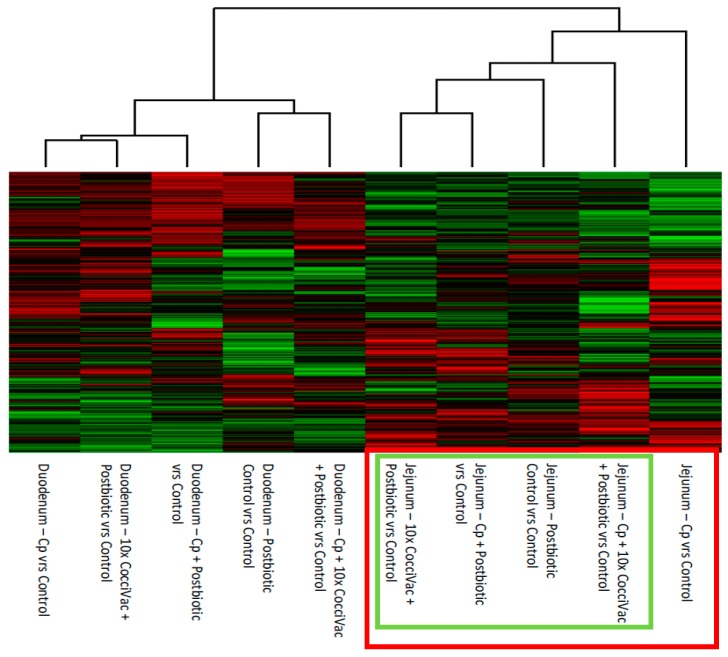
Heatmap and Clustering of Treatment Kinome Profiles Relative to Control Kinome Profiles. The raw kinome signal from the peptide array was input into the custom software package PIIKA 2. PIIKA 2 combines the biological replicates for each treatment and tissue, normalizes the data, and generates a representative kinome profile. Here, the profiles for each treatment group are compared to the kinome profile of control groups. The resulting kinome profiles are then compared for relative similarity, and a heatmap shows the relative phosphorylation of each peptide on the array for a given treatment group relative to control. Cp = *C. perfringens*.

A heatmap was also generated representing the kinome profiles of the treatment/tissue combinations relative to the control kinome profiles for each respective tissue (Figure 2). This analysis allows one to focus only on the changes in phosphorylation due to the experimental conditions. Duodenum again showed little differentiation between groups. Jejunum samples clustered based on postbiotic treatment (green box) with the non-postbiotic-treated group clustering furthest apart (far right column). Within the postbiotic-treated samples, the most separated column was the NE challenge (*C. perfringens* plus Coccivac) and postbiotic-treated group, suggesting this kinome profile was the most distinct compared to the other postbiotic-treated groups.

### 3.3. Postbiotic, C. perfringens and the Combination Each Uniquely Affected the Jejunum

**Figure 3:**
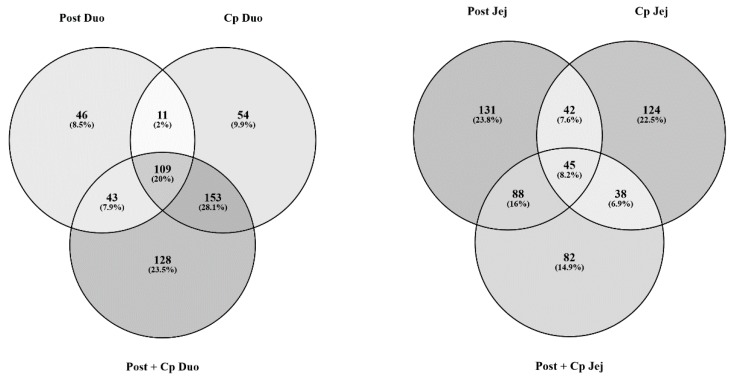
Venn Diagrams of differentially phosphorylated peptides in the postbiotic (Post), *C. perfringens* challenge (Cp) and postbiotic plus *C. perfringens* challenge (Post plus Cp) groups in duodenal (Duo) and jejunal (Jej) tissues. Peptides statistically significantly differentially phosphorylated (*p* < 0.05) from their respective control tissue were input into a Venn diagram-generating program Venny (http://bioinfogp.cnb.csic.es/tools/venny/).

For each peptide, representing a kinase target sequence, a fold change and *p*-value were generated. Peptides with a *p*-value of ≤ 0.05 were considered statistically significantly differentially phosphorylated relative to control. These statistically significant peptides were used to generate Venn diagrams in order to compare the effects of the postbiotic to *C. perfringens* inoculation in the jejunum and the duodenum (postbiotic, *C. perfringens*-challenged, and postbiotic plus *C. perfringens* challenge). Generally, the largest proportions of statistically significantly differentially phosphorylated peptides in the duodenal tissue (Duo) were shared between the groups, particularly between the *C. perfringens* challenge and the postbiotic plus *C. perfringens* challenge groups. This is consistent with the heatmaps shown previously. For the jejunal tissues, the largest proportions of statistically significantly differentially phosphorylated peptides were unique to each treatment group. As there was a large proportion of overlap in significant peptides between groups in the duodenum samples (Figure 3), and the duodenum did not cluster clearly by postbiotic treatment (Figure 1), it was determined that the main effect of the postbiotic occurred in the jejunum rather than the duodenum. Thus, subsequent analysis was conducted on the jejunum data to determine these observed effects.

**Figure 4:**
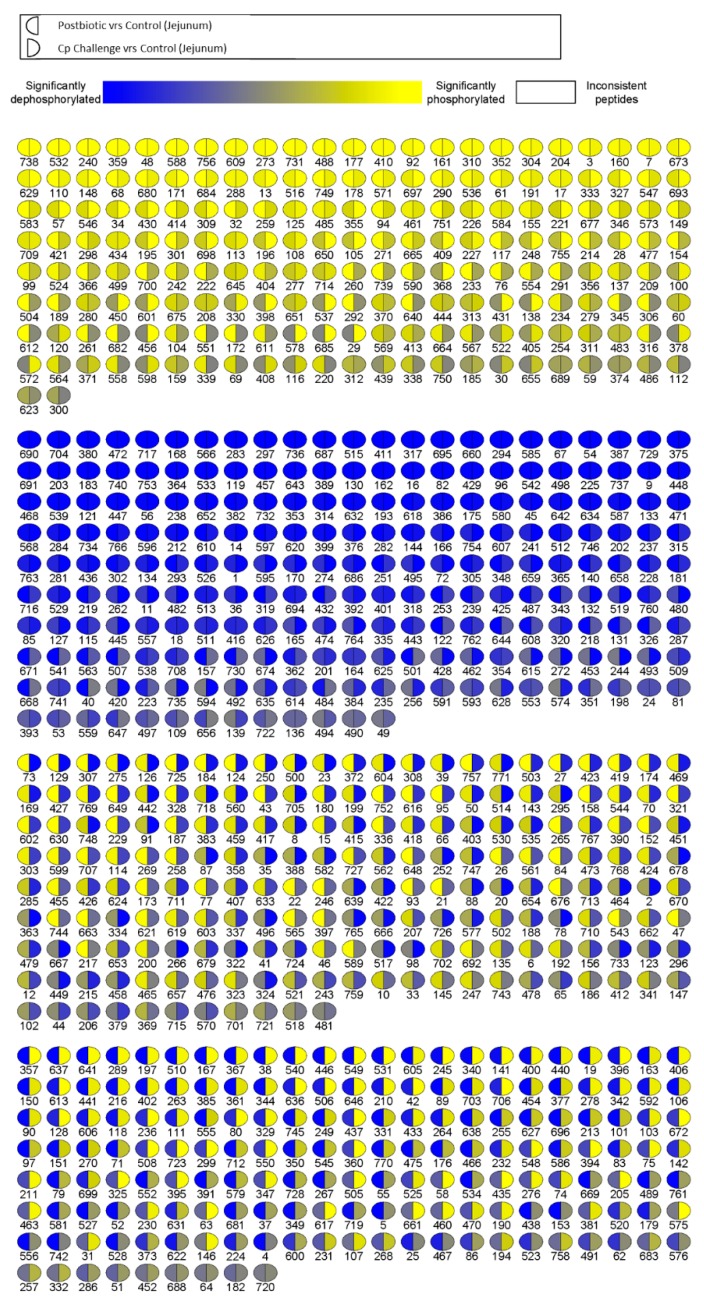
Peptide Phosphorylation Differentially Affected by Treatment with Postbiotic or *C. perfringens* Challenge. Each spot represents a peptide on the array which corresponds to a kinase recognition site. Each number represents a peptide, and the identities of each peptide can be found in Supplementary Table 1. The left half of each spot shows the differential phosphorylation status relative to control birds for postbiotic treatment in the jejunum. The right side of each spot shows the phosphorylation status relative to control birds for *C. perfringens* challenge in the jejunum.

A visual representation of *p*-values for each peptide represented on the array for postbiotic (relative to control) and *C. perfringens* (relative to control) groups is shown in Figure 4. The numbers below each spot represent peptides on the array, and their identities can be found in Supplementary Table S1. In this figure, each spot is divided in half, with the left side representing the postbiotic effects and the right side the *C. perfringens* effects. Blue represents a scale of significance showing decrease in phosphorylation relative to control, while yellow represents a scale of significance showing an increase in phosphorylation relative to control. The brightness of the color corresponds to the *p*-value or significance of the fold-change, with the brighter colors being the most significantly differentially phosphorylated relative to control and the more grey colors being less significant. The peptides are arranged into four blocks based on the comparison of the two sides of the spots. The first block is all yellow, meaning the peptides in this section are phosphorylated relative to control for both groups. The second block is blue, meaning the peptides in this section are less phosphorylated relative to control for both treatment groups. The third block shows peptides that were phosphorylated relative to control in the postbiotic group (left side of the circle) and dephosphorylated relative to control in the *C. perfringens* group (right side of the circle). The fourth block shows peptides that were less phosphorylated relative to control in the postbiotic group (left side of the circle) and phosphorylated relative to control in the *C. perfringens* group (right side of the circle). The results presented in Figure 4 show that there were a large proportion of peptides being differentially phosphorylated in opposite directions between the two groups (i.e., Blocks 3 and 4). Thus, jejunal tissues from the postbiotic group and the *C. perfringens* challenge group were responding distinctly to these two experimental conditions. 

### 3.4. Postbiotic and C. perfringens Uniquely Impact the Immune System While the Combination Shows Less Immune Responses

STRING is an online protein–protein interaction database that can provide useful insight into signal transduction pathways and biological processes from transcriptomic and kinomic data [20]. Here, we input protein identifiers for statistically significantly differentially phosphorylated peptides within our kinome dataset. In order to determine the effect of the metabolite on a pathogenic challenge, we focused on three groups: Postbiotic-treated, *C. perfringens*-challenged, and postbiotic plus *C. perfringens* challenge. Our analysis centered on those peptides that were uniquely differentially phosphorylated in one group but not observed as significant in the other two, i.e., the non-overlapping peptides within the Venn diagram (Figure 3). Focusing on this group of peptides allowed us to determine the physiological effects of each experimental group uniquely by removing the overlapping signal to the greatest extent possible. 

One of the STRING outputs was gene ontology (GO) biological process terms. The top 20 biological processes enriched between the three groups from their unique protein lists, postbiotic, *C. perfringens* challenge, and postbiotic plus *C. perfringens* challenge can be seen in Table 4, Table 5, and Table 6, respectively. While the biological processes may overlap between the tables, the input peptides had to be unique in order to be included in the analysis. Therefore, the same terms may appear between groups, but they were enriched in different ways between the groups. This indicates that the various biological processes enriched were uniquely influenced between the experimental groups. 

Highlighted in bold type are the terms related to immune signaling. All of the biological processes highlighted in the unique to postbiotic table (Table 4) also appear in the unique to *C. perfringens* challenge table (Table 5), but it is important to note that the peptides used to generate these tables were unique to each group. Interestingly, the combination postbiotic plus *C. perfringens* challenge table does not have the same immune related biological processes enriched in the top 20 results. This indicates that the overlap of the two treatments, the postbiotic and *C. perfringens* challenges, likely imparted their own effects in jejunal tissue, which, when brought together, did not synergize to enrich new immune related biological processes. In fact, there is a significant reduction in the number of peptides and KEGG pathways affected uniquely by the combination of postbiotic plus *C. perfringens*, highlighted by the reduction in statistically significantly differentially phosphorylated peptides and only 14 significant KEGG pathways unique to the combination. Rather, the postbiotic induced responses counteracted the *C. perfringens* induced responses, leaving little immune related signaling remaining.

### 3.5. Pathway Analysis Shows the Postbiotic Alters Innate Immunity While C. perfringens Alters Adaptive Immunity and the Combination Results in Reduced Signaling Responses

Similar to the GO biological process analysis above, we considered the KEGG pathways output from the STRING database. The top 20 KEGG pathways enriched from the input of peptides unique to each group (postbiotic, *C. perfringens* challenge and postbiotic plus *C. perfringens* challenge) can be seen in Table 7, Table 8, and Table 9, respectively. The proteins that were statistically significantly differentially phosphorylated uniquely in each experimental group were input into STRING and used to generate tables of KEGG pathways. While the KEGG pathways may overlap between the tables, the peptide members of the pathways had to be unique in order to be included in the analysis. Therefore, the same terms may appear between groups, but they are being enriched in different ways between the groups. This indicates that the various KEGG pathways enriched were being influenced in different ways between the groups. Highlighted in bold type are the pathways related to immune response. 

From the results above, we decided to analyze, in more detail, the top KEGG pathways generated from the unique to postbiotic peptides and unique to *C. perfringens* challenge peptides. These pathways were PI3K-Akt for the postbiotic group and the insulin signaling pathway for the *C. perfringens* challenge group. Table 10 contains the peptide members, fold change, and *p*-value data for the PI3K-Akt signaling pathway represented on the peptide array. The statistically significant fold change and *p*-values are in bold type. It is apparent that a large proportion of the peptides showed decreased phosphorylation relative to control in the postbiotic column. This is especially noteworthy in the critical signaling proteins, nuclear factor kappa-light-chain-enhancer of activated B cells (NFκB) at site S337 (which would lead to deactivation [22]), tuberous sclerosis complex 2 (TSC2) (which is involved in mammalian target of rapamycin (mTOR) deactivation [23]), protein kinase B (Akt) site T308 (which would lead to deactivation [24]), and protein kinase C alpha (PRKCA) (the decreased phosphorylation of which regulates translocation to pericentrion [25]). 

Table 11 contains the peptide members, fold change, and *p*-value data for the insulin signaling pathway represented on the peptide array. The statistically significant fold change and *p*-values are in bold type. Of note is that the statistically significant peptides in Table 11 affected by *C. perfringens* challenge map onto “response to cytokine” (GO biological processes), “signaling by interleukins” (reactome pathways) based on STRING analysis (data not shown). This is likely due to the immune activation of *C. perfringens*. As can be observed in Table 11, all peptides significantly affected by *C. perfringens* were either not affected or are inversely affected by the postbiotic.

## 4. Discussion

The goal of this study was to determine the mode of action of an in-water postbiotic product and its effects on gastrointestinal challenge in broiler chickens. Besides feeding the postbiotic itself, various challenges were tested, including *C. perfringens* inoculation, 10 X Coccivac inoculation, and *C. perfringens* plus Coccivac inoculation as a model for NE. We also considered two segments of the gut, the duodenum and the jejunum, to determine the main location of activity of the postbiotic. 

Between two replicate trials compared to challenge groups, the addition of the postbiotic showed an improved weight gain, decreased *C. perfringens* colony counts, decreased lesions scores and decreased mortality (Table 2 and Table 3). The postbiotic alone did not improve weight gain compared to control, non-challenged, birds. This may be due to the immune modulation we observed with the postbiotic, engaging certain aspects of the immune system, potentially affecting growth. However, this immune modulation also helped to maintain growth and health during an infectious challenge. 

The comparison of experimental groups, including control groups separately, showed that the jejunum had the most consistent response to the postbiotic compared to the duodenum (Figure 1). This jejunum response was further supported when we removed the control kinase activity signal from the data by comparing all kinome profiles to their respective non-treated, tissue matched controls (Figure 2). The jejunum had a consistent step-wise clustering of samples with the group not treated with postbiotic clustering as the most distinct. The group without the postbiotic (jejunum inoculated with *C. perfringens*) did not display responses as similar to the other groups and thus was clustered furthest out (to the right side of the figure). The duodenum, however, showed a clustering of challenge and metabolite without a clear pattern. Our interpretation is that the duodenum is less responsive to the postbiotic. Thus, the predominate kinome profile signal comes from the response to challenge. 

To verify further this differential response to the postbiotic between the jejunum and the duodenum, we compared the peptides that were statistically differentially phosphorylated between postbiotic treatment, *C. perfringens* challenge and the combination of postbiotic plus *C. perfringens* challenge in the duodenum and the jejunum, separately (Figure 3). These results confirmed what we had observed from the heatmap and cluster data; there was a significant amount of overlap between groups in the duodenum. Fully 20% of the differentially phosphorylated peptides were common between all three experimental groups. In addition, 28.1% were common between the *C. perfringens* challenge and the *C. perfringens* challenge plus postbiotic group. Combined, this represented nearly half of all differentially phosphorylated peptides in the data set. In contrast, the jejunum displayed distinct responses between each experimental group. There was relatively little overlap between the different groups in the jejunum; 61.2% of all differentially phosphorylated peptides were unique to one of the three groups. These results show that the jejunum was highly responsive to each of the experimental interventions. Based on the above results, subsequent analysis was conducted on the jejunum and three of the experimental groups, postbiotic, *C. perfringens* challenge and postbiotic plus *C. perfringens* challenge.

In order to visualize the differences between postbiotic-treated and *C. perfringens*-challenged kinome profiles, we generated a visualization map of the peptide phosphorylation (Figure 4). This map includes all peptides on the array, and each spot is split between postbiotic-treated (left) and *C. perfringens*-challenged (right). The map shows that roughly half of the peptides were differentially phosphorylated. In fact, they were phosphorylated in opposite directions between the two groups (i.e., are yellow on one half and blue on the other half), while half were similarly phosphorylated (i.e. are all yellow or all blue). It is important to note that not all of these events are statistically significant; the colors show the significance and direction of phosphorylation (increased or decreased relative to control). The obvious difference between these two groups agrees with the heatmap, where the *C. perfringens* challenge is the furthest clustered group. 

Since the majority of the differentially phosphorylated peptides were unique to each of the three groups, we used these unique peptide lists to conduct biological function analysis using the online STRING protein–protein interaction database [20]. The postbiotic peptide list generated a number of immune GO biological processes (Table 4, bold type). This indicated that the postbiotic had an impact on immune responses within the jejunal tissue relative to control. Conducting the same analysis with the *C. perfringens* challenge unique peptide list generated a larger number of immune related GO biological process top hits (Table 5, bold type). Ten of the top 20 most significant biological processes were immune related. As *C. perfringens* is a pathogenic bacterium that can elicit significant gastrointestinal pathology in broiler chickens, it is consistent with previous data that *C. perfringens* would generate a significant immune response [26,27,28]. When the same analysis was done considering the unique peptides from the *C. perfringens* plus postbiotic group, none of the top 20 biological processes were immune response related (Table 6). The combination of *C. perfringens* and the postbiotic results in unique phosphorylation events that were not related to immunity. Given Figure 4, where we see that many of the phosphorylation events elicited by *C. perfringens* and the postbiotic were in opposite directions, the combination appears to cancel out the others responses, thus resulting in a lack of unique immune signaling. This provides intriguing evidence that, coupled with an immune inducing pathogen, the postbiotic results in a homeostatic immune modulation. 

A more detailed analysis of the significant peptide phosphorylation involves incorporating them into specific signal transduction pathways. The STRING database organized the peptides into KEGG pathways and generated a significance value for each pathway. Table 7 displays the top 20 KEGG pathways generated from the unique peptide list in the postbiotic-treated jejunum. In Table 7, there are a number of immune pathways highlighted in bold type, several of which including the PI3K-Akt signaling pathway, the toll-like receptor signaling pathway, and the tumor necrosis factor (TNF) signaling pathway can be classified as innate immune pathways. Likewise in Table 8 are the KEGG pathways generated from the unique peptides from the *C. perfringens* challenge jejunum. Here, the immune pathways were predominately-adaptive immune pathways including the T cell receptor signaling pathway, natural killer cell mediated cytotoxicity, and the Fc epsilon receptor I (Fc epsilon RI) signaling pathway. Given that the postbiotic generated responses within innate immune pathways and *C. perfringens* generated responses within adaptive immune pathways, this explains why we see GO biological processes related to immune response but with different peptide members eliciting those responses. One set was innate, and one set was adaptive. The combined postbiotic and *C. perfringens* challenge group unique peptides generated substantially fewer KEGG pathways—14 total pathways. While there was some overlap in pathways from the previous KEGG lists, including insulin signaling and PI3K-AKT signaling pathways, there were also unique pathways such as the chemokine signaling pathway. Given the smaller number of pathways and the fewer peptides within those pathways, it appears that the combination muted the response of the two individual stimulating experimental treatments. Perhaps putting the birds in a more hemostatic but certainly less immunologically active state. 

Finally, it was of interest that the top KEGG pathway in the postbiotic dataset was the PI3K-Akt signaling pathway, which did not show up as a top pathway in the *C. perfringens* challenge data, while the insulin signaling pathway was the top pathway in the *C. perfringens* challenge data and did not show up in the postbiotic dataset. We have presented the individual peptides and their phosphorylation states in these pathways for each experimental group in Table 10 and Table 11. It can be observed from these tables that PI3K-Akt members were predominately dephosphorylated in the postbiotic treatment group, and these corresponded to deactivating events in critical proteins including NFκB [22], Akt [24], PRKCA [25], and likely TSC2 [23] (though the function of this site is not well characterized) (Table 10). The insulin signaling pathway as described in the KEGG database is a pathway that incorporates a variety of immune and metabolic responses, including mitogen-activated protein kinase (MAPK) signaling, glucose metabolism, apoptosis and phosphatidyl inositol signaling [21]. When the statistically significant proteins affected by *C. perfringens* (Table 11) were put into the STRING database, their function was mapped to immune related responses including “response to cytokine” (GO biological processes) [29] and “signaling by interleukins” (reactome pathways) [30]. The postbiotic-affected proteins mapped to generic cellular signal transduction responses. This result indicates that *C. perfringens* had an impact on innate immune responses not observed in the postbiotic treatment group.

## 5. Conclusions

In summary, the postbiotic studied here imparts an immunomodulatory effect on jejunal tissue in broilers. The postbiotic alone modulates the activation of the innate immune response, and when combined with a pathogenic challenge of *C. perfringens*, it inhibited the activation of standard *C. perfringens* immune responses. This may be an important intervention in maintaining a healthy gut, especially considering the restricted use of in-feed antibiotics, which are believed to impart an anti-inflammatory effect in the gut [31]. The gut needs to maintain both a state of tolerance (to commensal microbes) as well as a readiness to respond (to pathogenic organisms). This balance must be maintained in order to allow for optimum growth efficiency and health.

## 6. Patents

US UTL (Prioritized) P-A S-N 16/220,416 for Probiotics and Fermentation Metabolites for the Prevention and Treatment of Disease Conditions in Animals (Stinson Ref. No.: 3506155-0004).

WIPO P-A No. PCT/US2018/065683 for Probiotics and Fermentation Metabolites for the Prevention and Treatment of Disease Conditions in Animals.

## Figures and Tables

**Table 1:** Experimental Treatment Groups.

Treatment	Groups	Body Weights	Coccidial Challenge	*C. perfringens*	Lesion Score
T1	Negative Control	Day 1, 14, 21	No	No	Day 21
T2	Neg control + postbiotic	Day 1, 14, 21	No	No	Day 21
T3	10X Coccivac	Day 1, 14, 21	Day 14	No	Day 21
T4	10X Coccivac + *C. perfringens*	Day 1, 14, 21	Day 14	Day 17, 18, and 19	Day 21
T5	Positive *C. perfringens* control	Day 1, 14, 21	No	Day 17, 18, and 19	Day 21
T6	Coccivac + *C. perfringens* + postbiotic	Day 1, 14, 21	Day 14	Day 17, 18, and 19	Day 21
T7	Coccivac + postbiotic	Day 1, 14, 21	Day 14	No	Day 21
T8	*C. perfringens* + postbiotic	Day 1, 14, 21	No	Day 17, 18, and 19	Day 21

**Table 2:** Bird Trial 1.

Treatments	cfu of *C. perfringens* (× 10^5^)	Weight Gain (grams)	Mean Lesion Score	Mortality (Deaths/Total)
Control	0.03 ± 0.003 ^a^	803 ± 17 ^a^	0.13 ± 0.01 ^a^	0/24
Postbiotic only	0.02 ± 0.001 ^a^	729 ± 21 ^b^	0.46 ± 0.01 ^a^	2/24
Coccivac	0.21 ± 0.04 ^a^	756 ± 14 ^b^	1.63 ± 0.11 ^b^	0/25
Coccivac + postbiotic	0.31 ± 0.04 ^a^	708 ± 20 ^c^	0.56 ± 0.02 ^a^	1/25
*C. perfringens* only	14.0 ± 1.51 ^b^	695 ± 22 ^d^	1.00 ± 0.03 ^b^	2/25
*C. perfringens* + postbiotic	9.5 ± 1.21 ^c^	688 ± 23 ^d^	0.70 ± 0.02 ^c^	2/25
Coccivac + *C. perfringens*	6.2 ± 1.71 ^c^	697 ± 17 ^d^	0.52 ± 0.01 ^c^	3/25
Coccivac + *C. perfringens* + postbiotic	1.7 ± 0.04 ^d^	781 ± 11 ^a^	1.00 ± 0.02 ^b^	1/25

^a,b,c^: Numbers with different superscripts within the same column are statistically significantly different from each other.

**Table 3:** Bird Trial 2.

Treatments	cfu of *C. perfringens* (× 10^5^)	Weight Gain (Grams)	Mean Lesion Score	Mortality (Deaths/Total)
Control	0.01 ± 0.002 ^a^	857 ± 31 ^a^	0.0 ± 0.0 ^a^	0/25
Postbiotic only	0.01 ± 0.001 ^a^	779 ± 17 ^b^	0.0 ± 0.0 ^a^	0/25
Coccivac	0.02 ± 0.002 ^a^	766 ± 22 ^b^	0.8 ± 0.01 ^b^	0/25
Coccivac + postbiotic	0.01 ± 0.002 ^a^	758 ± 27 ^b^	0.2 ± 0.003 ^c^	1/25
*C. perfringens* only	5.9 ± 1.06 ^b^	647 ± 31 ^c^	1.35 ± 0.11 ^d^	4/25
*C. perfringens* + postbiotic	2.3 ± 0.97 ^c^	725 ± 24 ^b^	0.22 ± 0.03 ^c^	1/25
Coccivac + *C. perfringens*	7.4 ± 1.32 ^b^	664 ± 33 ^c^	1.89 ± 0.41 ^d^	5/25
Coccivac + *C. perfringens* + postbiotic	0.5 ± 0.003 ^d^	795 ± 27 ^a^	0.41 ± 0.02 ^e^	0/25

^a,b,c^: Numbers with different superscripts within the same column are statistically significantly different from each other.

**Table 4:** Top 20 gene ontology (GO) biological processes enriched from unique peptides in the postbiotic-treated jejunum (compared to *C. perfringens* challenge and postbiotic plus *C. perfringens* challenge groups).

Biological Process	Numberof Proteins	*p*-Value (False Discovery Rate)
cellular response to chemical stimulus	36	8.62 × 10^12^
cellular response to organic substance	32	3.00 × 10^11^
phosphorylation	25	9.43 × 10^11^
transmembrane receptor protein tyrosine kinase signaling pathway	21	1.13 × 10^10^
enzyme linked receptor protein signaling pathway	23	2.10 × 10^10^
protein phosphorylation	21	1.03 × 10^9^
regulation of signaling	34	7.35 × 10^9^
regulation of response to stimulus	36	6.90 × 10^8^
**regulation of immune response**	**19**	**6.90 × 10^8^**
phosphorus metabolic process	27	7.12 × 10^8^
response to organic substance	31	7.12 × 10^8^
regulation of cell communication	33	9.58 × 10^8^
**regulation of immune system process**	**23**	**1.57 × 10^7^**
phosphate-containing compound metabolic process	26	1.94 × 10^7^
cell surface receptor signaling pathway	27	6.22 × 10^7^
regulation of signal transduction	29	6.58 × 10^7^
response to chemical	36	9.23 × 10^7^
**positive regulation of immune system process**	**17**	**2.70 × 10^6^**
**innate immune response**	**18**	**2.70 × 10^6^**
single-organism metabolic process	37	4.38 × 10^6^

Proteins statistically significantly differentially phosphorylated uniquely in the jejunal tissue samples from the postbiotic treatment group were pulled out of the array data and input into STRING for analysis. The resulting table of biological processes contained 248 terms enriched in the data. The top 20 by false discovery rate are shown in this table. Bold type indicates processes related to immune response.

**Table 5:** Top 20 GO biological processes enriched from unique peptides in the *C. perfringens* challenge jejunum (compared to postbiotic and postbiotic plus *C. perfringens* challenge groups).

Biological Process	Number of Proteins	*p*-Value (False Discovery Rate)
**regulation of immune response**	**20**	**3.90 × 10^9^**
protein metabolic process	38	1.15 × 10^8^
cellular protein metabolic process	35	1.31 × 10^8^
positive regulation of metabolic process	35	1.80 × 10^8^
regulation of phosphate metabolic process	24	1.80 × 10^8^
intracellular signal transduction	26	1.80 × 10^8^
**defense response**	**23**	**2.02 × 10^8^**
regulation of phosphorylation	22	2.02 × 10^8^
**positive regulation of immune response**	**16**	**2.02 × 10^8^**
**immune response-regulating signaling pathway**	**15**	**2.44 × 10^8^**
regulation of protein modification process	24	2.47 × 10^8^
protein phosphorylation	18	3.17 × 10^8^
**innate immune response**	**19**	**3.93 × 10^8^**
**regulation of immune system process**	**22**	**4.69 × 10^8^**
**activation of immune response**	**14**	**5.11 × 10^8^**
response to nitrogen compound	18	8.11 × 10^8^
**immune response-activating signal transduction**	**13**	**1.35 × 10^7^**
**Fc receptor signaling pathway**	**11**	**1.35 × 10^7^**
**Fc-epsilon receptor signaling pathway**	**10**	**1.46 × 10^7^**
regulation of protein phosphorylation	20	1.60 × 10^7^

Proteins statistically significantly differentially phosphorylated uniquely in the jejunal tissue samples from the *C. perfringens* challenge treatment group were pulled out of the array data and input into STRING for analysis. The resulting table of biological processes contained 258 terms enriched in the data. The top 20 by false discovery rate are shown in this table. Bold type indicates processes related to immune response.

**Table 6:** Top 20 GO biological processes enriched from unique peptides in the postbiotic plus *C. perfringens* challenge jejunum (compared to postbiotic and *C. perfringens* challenge groups).

Biological Process	Number of Proteins	*p*-Value (False Discovery Rate)
protein autophosphorylation	11	9.87 × 10^9^
enzyme linked receptor protein signaling pathway	17	5.74 × 10^8^
transmembrane receptor protein tyrosine kinase signaling pathway	15	1.05 × 10^7^
regulation of cellular protein metabolic process	21	1.08 × 10^5^
regulation of intracellular signal transduction	17	1.08 × 10^5^
positive regulation of kinase activity	11	1.18 × 10^5^
positive regulation of lipid metabolic process	7	1.18 × 10^5^
intracellular signal transduction	18	4.55 × 10^5^
regulation of protein kinase activity	12	4.55 × 10^5^
response to external stimulus	18	4.88 × 10^5^
positive regulation of catalytic activity	16	4.88 × 10^5^
regulation of multicellular organismal process	20	4.88 × 10^5^
positive regulation of molecular function	17	6.06 × 10^5^
peptidyl-tyrosine phosphorylation	7	6.85 × 10^5^
protein phosphorylation	12	0.000123
regulation of cellular component biogenesis	11	0.000123
regulation of protein modification process	16	0.000124
regulation of protein phosphorylation	14	0.000128
positive regulation of intracellular signal transduction	12	0.000132
axon guidance	9	0.000151

Proteins statistically significantly differentially phosphorylated uniquely in the jejunal tissue samples from the postbipotic plus *C. perfringens* challenge treatment group were pulled out of the array data and input into STRING for analysis. The resulting table of biological processes contained 269 terms enriched in the data. The top 20 by false discovery rate are shown in this table. Bold type indicates processes related to immune response.

**Table 7:** Top 20 KEGG pathways enriched from unique peptides in the postbiotic jejunum (compared to *C. perfringens* challenge and postbiotic plus *C. perfringens* challenge).

KEGG Pathway	Number of Proteins	*p*-Value (False Discovery Rate)
**PI3K-Akt signaling pathway**	**14**	**6.90 × 10^9^**
**Fc gamma R-mediated phagocytosis**	**9**	**6.90 × 10^9^**
Epstein–Barr virus infection	11	1.56 × 10^8^
Pancreatic cancer	7	2.58 × 10^7^
Non-alcoholic fatty liver disease (NAFLD)	9	3.08 × 10^7^
Ras signaling pathway	10	4.99 × 10^7^
Toxoplasmosis	8	4.99 × 10^7^
Influenza A	9	5.57 × 10^7^
Osteoclast differentiation	8	7.90 × 10^7^
Hepatitis C	8	9.40 × 10^7^
Measles	8	9.40 × 10^7^
Pathways in cancer	11	9.70 × 10^7^
Hepatitis B	8	1.42 × 10^6^
VEGF signaling pathway	6	2.15 × 10^6^
**Toll-like receptor signaling pathway**	**7**	**2.40 × 10^6^**
**TNF signaling pathway**	**7**	**3.14 × 10^6^**
**Fc epsilon RI signaling pathway**	**6**	**3.49 × 10^6^**
Adipocytokine signaling pathway	6	3.61 × 10^6^
Prolactin signaling pathway	6	4.08 × 10^6^
Adherens junction	6	4.19 × 10^6^

Proteins statistically significantly differentially phosphorylated uniquely in the jejunal tissue samples from the postbiotic treatment group were pulled out of the array data and input into STRING for analysis. The resulting table of KEGG pathways contained 92 terms enriched in the data. The top 20 by false discovery rate are shown in this table. Highlighted in bold type are the pathways related to immune response.

**Table 8:** Top 20 KEGG pathways enriched from unique peptides in the *C. perfringens* challenge jejunum (compared to postbiotic and postbiotic plus *C. perfringens* challenge).

KEGG Pathway	Number of Peptides	*p*-Value (False Discovery Rate)
**Insulin signaling pathway**	**8**	**4.18 × 10^6^**
**T cell receptor signaling pathway**	**7**	**5.66 × 10^6^**
Neurotrophin signaling pathway	7	1.14 × 10^5^
**Natural killer cell mediated cytotoxicity**	**7**	**1.36 × 10^5^**
Focal adhesion	8	2.00 × 10^5^
Prostate cancer	6	2.00 × 10^5^
Estrogen signaling pathway	6	2.93 × 10^5^
Pathways in cancer	9	4.47 × 10^5^
Glioma	5	6.17 × 10^5^
**Fc epsilon RI signaling pathway**	**5**	**8.24 × 10^5^**
Osteoclast differentiation	6	8.79 × 10^5^
Prolactin signaling pathway	5	8.79 × 10^5^
Hepatitis C	6	0.000102
ErbB signaling pathway	5	0.000183
MAPK signaling pathway	7	0.000416
Endometrial cancer	4	0.000436
Viral carcinogenesis	6	0.000532
Non-small cell lung cancer	4	0.000532
FoxO signaling pathway	5	0.000803
Measles	5	0.00108

Proteins statistically significantly differentially phosphorylated uniquely in the jejunal tissue samples from the *C. perfringens* challenge group were pulled out of the array data and input into STRING for analysis. The resulting table of KEGG pathways contained 69 terms enriched in the data. The top 20 by false discovery rate are shown in this table. Highlighted in bold type are the pathways related to immune response.

**Table 9:** KEGG pathways enriched from unique peptides in the postbiotic plus *C. perfringens* challenge jejunum (compared to postbiotic and *C. perfringens* challenge).

KEGG Pathway	Number of Proteins	*p*-Value (False Discovery Rate)
AMPK signaling pathway	7	4.40 × 10^6^
Acute myeloid leukemia	5	3.23 × 10^5^
**Chemokine signaling pathway**	**6**	**0.000441**
**Insulin signaling pathway**	**5**	**0.00125**
**PI3K-Akt signaling pathway**	**6**	**0.00685**
Leukocyte transendothelial migration	4	0.00685
Pathways in cancer	6	0.00685
mTOR signaling pathway	3	0.0146
ErbB signaling pathway	3	0.0329
Endocytosis	4	0.0329
**Fc gamma R-mediated phagocytosis**	**3**	**0.0329**
Regulation of actin cytoskeleton	4	0.0397
Proteoglycans in cancer	4	0.0405
Thyroid cancer	2	0.0405

Proteins statistically significantly differentially phosphorylated uniquely in the jejunal tissue samples from the postbiotic plus *C. perfringens* challenge treatment group were pulled out of the array data and input into STRING for analysis. The resulting table of KEGG pathways is shown above. Less than 20 significant pathways (false discovery rate) were generated. Highlighted in bold type are the pathways related to immune response.

**Table 10:** Members of the PI3K-Akt signaling pathway showing differential phosphorylation in jejunal tissue.

Protein Name	UniProt ID	p-Site	Postbiotic vs. Control		*C. perfringens* vs. Control	
			Fold-Change	*p*-Value	Fold-Change	*p*-Value
CHUK	O15111	S180	**−1.02651**	**0.00234**	1.01325	0.13513
PDPK1	O15530	Y376	**−1.02194**	**0.00604**	1.00729	0.24518
EGFR	P00533	Y1069	**1.01442**	**0.04356**	−1.00779	0.27386
HRAS	P01112	T35	**1.02607**	**0.00545**	−1.0091	0.16151
RAF1	P04049	S338	**1.01503**	**0.03641**	−1.00407	0.32867
CSF1R	P07333	Y809	**−1.01495**	**0.04428**	1.00672	0.28552
NGFR	P08138	S303	**−1.01773**	**0.0282**	1.01786	0.05842
MET	P08581	Y1356	1.01016	0.12976	**−1.02576**	**0.03017**
PDGFRB	P09619	Y857	−1.01021	0.18692	**1.03092**	**0.00766**
FGFR1	P11362	Y654	1.00115	0.45799	**−1.02374**	**0.01296**
ATF2	P15336	T69	**1.02311**	**0.00053**	−1.00301	0.34632
PDGFRA	P16234	Y1018	−1.01148	0.05865	**1.02937**	**0.00392**
PRKCA	P17252	S657	−1.00437	0.25123	**1.03971**	**0**
PRKCA	P17252	T638	**−1.02379**	**0.02882**	1.00343	0.38409
NFKB1	P19838	S337	**−1.02217**	**0.00403**	1.00684	0.27404
FGFR2	P21802	Y769	**1.02675**	**0.00114**	−1.00222	0.41273
RPS6KB1	P23443	T412	**1.02802**	**0.00423**	−1.0113	0.16029
AKT1	P31749	T308	**−1.01516**	**0.0485**	1.008	0.19793
AKT1	P31749	T479	**−1.01495**	**0.04114**	1.00149	0.44567
SYK	P43405	Y525	**1.01677**	**0.01122**	−1.00287	0.35294
TSC2	P49815	S1418	**−1.01828**	**0.00393**	**1.02324**	**0.01799**
GSK3B	P49841	S389	**−1.01759**	**0.04783**	1.00306	0.36676
GRB2	P62993	Y209	1.00065	0.46262	**−1.01717**	**0.02193**
RAC1	P63000	S71	**1.03273**	**0.00045**	−1.00615	0.21369
SOS1	Q07889	S1167	−1.00042	0.48408	**1.02671**	**0.03753**
SOS1	Q07889	S1193	−1.00949	0.12766	**1.02837**	**0.0056**
PRKAA1	Q13131	T183	**1.02076**	**0.01566**	−1.00035	0.48506
STK11	Q15831	T363	**1.02122**	**0.00098**	−1.00271	0.39339
CDC37	Q16543	S13	**−1.02066**	**0.00287**	1.00015	0.49454
AKT3	Q9Y243	S476	**−1.0311**	**0.00023**	1.01033	0.18851

Selection of peptides differentially phosphorylated between postbiotic group and *C. perfringens* challenge group in jejunal tissue. The UniProt ID identifies the protein, while the p-site identifies the specific phosphorylation target site on that protein. Fold-Change indicates the directionality of phosphorylation status for each treatment compared to control. The *p*-value is the measure of significance (α = 0.05). UniProt IDs and p-site correspond to human proteins for annotation purposes. Highlighted in bold type are the statistically significantly differentiated phosphorylated peptide results (*p* < 0.05).

**Table 11:** Members of the insulin signaling pathway showing differential phosphorylation in jejunal tissue.

Protein Name	UniProt ID	p-Site	Postbiotic vs. Control		*C. perfringens* vs. Control	
			Fold-Change	*p*-Value	Fold-Change	*p*-Value
SOCS3	O14543	Y204	−1.007	0.25196	1.03314	0.00195
PDPK1	O15530	Y376	**−1.02194**	**0.00604**	1.00729	0.24518
HRAS	P01112	T35	**1.02607**	**0.00545**	−1.0091	0.16151
RAF1	P04049	S338	**1.01503**	**0.03641**	−1.00407	0.32867
PRKCA	P17252	S657	−1.00437	0.25123	**1.03971**	**0**
PRKCA	P17252	T638	**−1.02379**	**0.02882**	1.00343	0.38409
PRKACA	P17612	T198	−1.00047	0.47688	**1.04907**	**0**
PRKACA	P17612	S140	**−1.0358**	**0.00013**	**1.01705**	**0.04542**
RPS6KB1	P23443	T412	**1.02802**	**0.00423**	−1.0113	0.16029
SHC1	P29353	Y427	**1.01952**	**0.00514**	−1.00286	0.3601
PKLR	P30613	Y564	**1.03513**	**0**	−1.00476	0.25834
PKLR	P30613	T556	**1.01773**	**0.01603**	−1.01034	0.08146
AKT1	P31749	T479	**−1.01495**	**0.04114**	1.00149	0.44567
AKT1	P31749	T308	**−1.01516**	**0.0485**	1.008	0.19793
SREBF1	P36956	S338	**−1.0149**	**0.02166**	1.00547	0.17584
PHKA2	P46019	S729	1.01244	0.17373	**−1.03329**	**0.02754**
PHKA1	P46020	Y549	1.00468	0.33438	**−1.03754**	**0.00368**
PHKA1	P46020	S972	**1.01286**	**0.04837**	**−1.01185**	**0.02087**
CRK	P46108	Y251	1.01333	0.07722	**−1.01651**	**0.02286**
TSC2	P49815	S1418	**−1.01828**	**0.00393**	**1.02324**	**0.01799**
GSK3B	P49841	S389	**−1.01759**	**0.04783**	1.00306	0.36676
HK2	P52789	Y461	**1.0372**	**0.00776**	−1.01701	0.15607
GRB2	P62993	Y209	1.00065	0.46262	**−1.01717**	**0.02193**
SOS1	Q07889	S1167	−1.00042	0.48408	**1.02671**	**0.03753**
SOS1	Q07889	S1193	−1.00949	0.12766	**1.02837**	**0.0056**
ACACA	Q13085	S1263	**−1.02066**	**0.01582**	1.00135	0.44476
PRKAA1	Q13131	T183	**1.02076**	**0.01566**	−1.00035	0.48506
MKNK1	Q9BUB5	T255	**−1.01817**	**0.01009**	1.00328	0.35474
AKT3	Q9Y243	S476	**−1.0311**	**0.00023**	1.01033	0.18851

Selection of peptides differentially phosphorylated between postbiotic group and *C. perfringens* challenge group in jejunal tissue. The UniProt ID identifies the protein, while the p-site identifies the specific phosphorylation target site on that protein. Fold-change indicates the directionality of phosphorylation status for each treatment compared to control. The *p*-value is the measure of significance (α = 0.05). UniProt IDs and p-site correspond to human proteins for annotation purposes. Highlighted in bold type are the statistically significantly differentiated phosphorylated peptide results (*p* < 0.05).

## Supplementary Materials

The following are available online at https://www.mdpi.com/2076-2607/7/8/268/s1, Table S1: Peptide Identifiers as Shown in Figure 4.
